# Annotations

**Published:** 1898-10-15

**Authors:** 


### ANNOTATIONS.

Lord Lister formally opened the Thompson-Yates Laboratories of Physiology and Pathology at Liverpool last week.

Mr. Jonathan Hutchinson has resumed his afternoon demonstrations at the Clinical Museum, 211, Great Portland Street, on Wednesdays, at four p.m. The demonstrations are open to all members of the medical profession.

According to the Paris correspondent of the *Lancet* Dr. Lucas Championnière has criticised very severely the treatment adopted by the surgeons in attendance upon the Prince of Wales, and expresses his astonishment that operative interference was not undertaken, seeing that the fragments of the patella were found soon after the accident to be so far apart.

A new Poor Law hospital has been opened at Mansfield by Lady Laura Ridding. The building consists of two wings, one in which the wards are situated, and the other devoted mainly to administrative purposes. The wards are situated on two storeys for the accommodation of 70 patients. The cost is estimated at between £10,000 and £11,000. The hospital, which bears the name of Victoria, has been erected by the Mansfield Board of Guardians.

Clinical lectures, free to students and practitioners, will be held at half-past three on the following Tuesdays, at the National Hospital for the Paralysed and Epileptic, Queen Square, W.C., as under: On October 18th, Dr. Buzzard, "Peripheral Neuritis"; October 25th, Dr. Ferrier, F.R.S., "Hemiplegia"; November 1st, Dr. Tooth, "Spinal Cord Localisation" (lantern demonstration); November 8th, Dr. Ormerod, "Muscular Atrophy"; November 15th, Sir W. Gowers, F.R.S., "Degenerative Diseases of the Nervous System in relation to Causation"; November 22nd, Dr. Taylor, "Cerebral and Spinal Paralysis in Children"; November 29th, Mr. Ballance, "A case of Fifth Nerve Neuralgia treated by Operation"; December 6th, Sir F. Semon, "On the probably Cortical Origin of some Laryngeal Paralyses"; December 13th, Mr. Victor Horsley, F.R.S., "Surgery of the Nervous System"; December 20th, Dr. Colman, "Syringomyelia."

Dr. James B. Russell has been appointed medical member of the Local Government Board for Scotland. For the last quarter of a century he has done admirable work in Glasgow as medical officer of health.

The memory of the late Greig Smith has been kept green by the erection of a Greig Smith Memorial Theatre at the Bristol Royal Infirmary. This was opened by Sir William MacCormac.

The London Post-Graduate Course, which arranges for lectures and demonstrations at various hospitals and institutions, commenced on October 10th. Full particulars are to be had from Dr. Fletcher Little, 32, Harley Street, W.

It is interesting to see that the *Lancet*, an organ recognised by the most exacting purists in the medical profession, has published among its abstracts of introductories the remarks made at the opening session of the Pharmaceutical Society and the Royal Veterinary College.

The new Director of the Natural History Museum is Professor Ray Lankester, Professor of Comparative Anatomy at Oxford. Previously he was Jodrell Professor at University College. By Professor Ray Lankester's appointment to South Kensington the chair at Oxford becomes vacant. Already there are a number of distinguished candidates seeking the post.

The annual banquet to 1,500 poor children of the Ragged School Union will on this occasion be held at the Guildhall, which the City Corporation have granted for the purpose. It is again proposed to send Christmas hampers to 5,000 crippled children; and Mr. Alderman Treloar is prepared to receive subscriptions for the purpose at 69, Ludgate Hill, E.C.

The Sixth International Otological Congress will be held in London on August 8th, 9th, 10th, 11th, and 12th, 1899. President—Dr. Urban Pritchard, Professor of Otology at King's College, London. The meetings will, by permission, be held at the Examination Hall of the Royal Colleges of Physicians and Surgeons, Victoria Embankment. The subject chosen for special discussion is "Indications for opening the Mastoid in Chronic Suppurative Otitis Media." A large and influential British Organisation Committee has been formed, the treasurer being Mr. A. E. Cumberbatch, and the hon. secretary Mr. Cresswell Baber. The International Otological Congress, which assembles every four years, met last in Florence, where a very successful gathering was held under the presidency of Professor Grazzi.

The Medical Society of London opened its session last Monday with an introductory address by Mr. E. Owen, the president, after which Dr. Allchin read a paper on "Some Considerations Preliminary to the Study of Dyspepsia." He pointed out that it was an error to look upon dyspepsia as a disorder of the stomach alone, for just as the whole of the alimentary canal is concerned in digestion, so could a much more extensive area than the stomach be concerned in the production of dyspepsia. He described the various symptoms of dyspepsia, and showed from how many different causes many of these symptoms might arise. Hence the difficulty which existed in diagnosing accurately the particular pathological condition which might exist in a given case. Nevertheless, probably all disorder of function was connected with structural change. The Lettsonian lectures will be delivered in February and March next by Dr. Samuel Gee, and the annual oration will be given by Mr. AlbanDoran.

### The Abernethian Society of St. Bartholomew's Hospital.

The annual opening address of the above society was given at the hospital on the evening of Thursday, October 6th, by Sir Thomas Smith, Bart. There was an enthusiastic audience of nearly 500 people. Sir Thomas described his earliest impressions when he entered the hospital some fifty years ago, and from those comparatively remote times he traced the progress which has taken place in its administration, practice, and teaching, and he dwelt upon the gradual improvement in the manners and morals of its students. After the address Sir Dyce Duckworth proposed a vote of thanks, which was seconded by Mr. Bowlby, and cordially responded to by those present.

### The Colour of Pure Water.

Those who have watched the Rhone pouring out of Lake Geneva must often have wondered as to the cause of the beautiful blue colour of its water. At the Congress of Hydrology and Climatology, which has just been held at Liege, it has been shown that blue is the natural colour of perfectly pure water, and that this tint, as seen in the Lake of Geneva, is neither due to reflection nor to the admixture of suspended particles, as has often been supposed. Very fine, colourless particles in suspension give rise to a yellowness which, combined with the natural blue, make certain waters, as in the lakes of Neuchatel and Constance, appear green, and when such green waters are again slightly tinted by admixture with a slight trace of a reddish, ferruginous mud, as occasionally happens, the red and the green tints neutralise each other, so that the water remains for the time absolutely colourless. Absolutely colourless water, then, is not pure.

### The Irish Dispensary Doctors.

The eloquent and timely address given by Surgeon McArdle on the opening of the winter session at St. Vincent's Hospital, Dublin, will, we hope, do something to help that most hard-working and well-deserving set of men, the Dispensary Medical Officers of Ireland. In accordance with the provisions of the new Local Government of Ireland Act, these officers are about to pass under the rule of bodies which are the outcome of an extended franchise, and Dr. McArdle has done well to urge that the moment should be seized to formulate for these bodies the views of the profession at large on the changes which are necessary to constitute and maintain the most efficient Poor Law medical service. The main grievances of the Poor Law medical officers in Ireland at the present time are as follows: (1) Their payment is inadequate; (2) there is no system of promotion in the service; (3) they have no fixed retiring allowances; and (4) the system of the red ticket distribution is a grave injustice. In regard to all these points, Dr. McArdle gave eloquent testimony as to the necessity for reform. Yet he is hopeful, and he places his trust in the justice and liberality of the new local bodies to whom such large powers are to be given. "Now I do not agree," he says, "with those who say that the new governing bodies will deal less fairly with their officers than those they supersede. My belief is that the consciousness of power, the necessary outcome of enfranchisement, is certain to elevate the tone and broaden the views of all who take part in the development of local government. Never in the history of mankind did the advent of liberty herald mental retrogression." We only hope that Surgeon McArdle may be right.

### Catching Trains.

Medical men make such constant use of railways, and matters of life and death so often hinge upon their catching trains, that they will do well to take note of a decision given last week in the Southwark County Court in regard to a claim against the London and South-Western Railway. The plaintiff had arrived at a station on the line in question more than two minutes before the time advertised for the departure of the train, and found the door locked in his face. The defendants' counsel relied upon the conditions published in the company's time tables: "The doors at the station may be closed five minutes before the departure of each train." His Honour remarked that nothing could be more fair and reasonable than the conditions named, and gave judgment for the defendants, with costs! We think, however, with the plaintiff, that if it is intended to take advantage of such conditions they should be given greater publicity than is the case, and should be acted on with consistency. For a railway company to keep a rule like that in its pocket for its own protection, and use it just when it likes, is manifestly unfair to the travelling public, while when applied to medical men rushing off to urgent cases it is sure to lead to embarrassment and perhaps to loss of life.

### Bacteriology and the Metropolitan Asylums Board.

At the meeting of the Metropolitan Asylums Board held last Saturday it was moved by Professor Smith, and carried, "That it be referred to the General Purposes Committee to consider the desirability or otherwise of the Board establishing a bacteriological laboratory for London, or of making other arrangements for providing bacteriological assistance in the diagnosis of infectious disease, with power to obtain the opinion of the several metropolitan sanitary authorities upon the subject." Thus we find another candidate in the field for the possession of the rate-supported laboratory, which will no doubt sooner or later have to be provided. We fail, however, to see the special fitness of the Asylums Board for the functions it seems anxious to assume, and there are many grave and serious reasons why that body should not be encouraged to intervene further in the sanitary administration of the metropolis. So far as its ambulance work and its treatment of such patients as gain access to its wards are concerned, no one can justly say anything against the way in which the Metropolitan Asylums Board has performed the duties laid upon it. But the position which it occupies in the sanitary administration of London is a very anomalous one; it is an intrusion of the poor law element into work with which the poor law has no concern at all, and although we may fairly admit that it has been as the result of the excellence of its work, coupled perhaps with the curious elasticity of its purse, that it has been allowed to usurp so many duties which belong properly to other bodies, it is impossible to avoid the conclusion that if the sanitary authorities of London are to occupy the position in regard to the health of London which they occupy in other towns, and which it was intended by the legislature that they should occupy here, it is most desirable that they should not delegate their duties to bodies in which they are unrepresented. That a great central health laboratory for London would be a great advantage we cannot doubt, but it should certainly be under the control either of the Government or of some combination of sanitary authorities—not under a body which, except through the slow and tedious proceedings of the Local Government Board, is under no control at all.

### Municipal Slaughter-Houses.

At a meeting of the London County Council which was held on Tuesday a matter was discussed which is of the greatest possible importance to all dwellers in the metropolis—namely, the closure of those numerous and widely scattered private slaughter-houses the existence of which is the main obstacle to proper and systematic meat inspection, and the erection in their place of well-built municipal abattoirs, in which not only may the whole process of slaughtering be carried out with the least possible nuisance to the public and the least possible cruelty to the animals, but in which facilities may be provided for the complete inspection of every carcase before it is put on the market as food for man. Needless to say objections were raised, and also needless to say these objections turned much more upon the closure of the private than the erection of the public slaughter-houses. The discussion was adjourned, and no doubt the butchers will make a brave fight for their privileges. From our point of view, however—that is from the point of view which regards the question merely as it affects the health of London—we cannot but feel that the Public Health Committee are in the right in proposing, as they do, that "as a first step towards insuring the proper inspection of meat, private slaughter-houses should cease to exist, and that butchers should in substitution be afforded such facilities as are necessary for the killing of animals in public slaughter-houses to be erected by the Council." The tendency of all discussion of this question is to drift away from the real point at issue, which is the inspection of the meat, and to concentrate itself on side issues, such as compensation and private rights, which after all are but details. This, however, should not be allowed for a moment. The proposal is brought forward, not in hostility to butchers, but as a great sanitary measure; and those who raise objections are bound to show how else complete inspection of meat can be obtained. At the same time we are bound to admit that the scheme of the County Council will be of but small avail for the protection of the metropolis unless at the same time some reform is instituted in the farcical inspection to which the foreign meat slaughtered at Deptford is subjected. The foreign meat trade and the English meat trade are the complements of one another, and unless the authorities which deal with both these trades advance hand in hand any improvement in the one will but tend to emphasise and aggravate the dangers of the other.

### The Mosquito Theory of Malaria.

So much has of late been discovered about malaria, and the presentment of the subject at the Edinburgh meeting of the British Medical Association, so far at least as concerns the natural history of the parasite, was so complete that we are now able to form a fair mental picture of the probable course of events when man becomes infected by the disease. The first thing, however, to bear in mind is that malaria is not primarily a disease of man, and that in the evolution of the parasite man has had no concern. A district may be thoroughly malarial, although man's footsteps may never have trodden upon its marshes, and the first time in the history of the world that man appears upon its virgin soil he may be at once affected with typical malaria, complete in all its symptoms, and punctual in its strange periodicity. The parasite evidently pre-exists fully developed, and we may be pretty sure that when man gets malaria his blood cells get a dose intended for some other form of protoplasm. Let us then, from the researches of Manson, Ross, and others, build up a mental picture of the vicissitudes which this strange organism may be supposed to undergo in the cycle of its life. Beginning with the blood just before the time when a rigor is due, it appears probable that the following events take place: (1) The malaria organism, having the form of a disc of protoplasm dotted with pigment, lies within a red blood corpuscle. (2) The formation within it of a number of spherules. (3) The discharge of these (spores?) into the liquor sanguinis. (4) The absorption of many of these spores by the leucocytes. Such as escape, however, enter into fresh red corpuscles, in which (5) they undergo various changes until they revert to the original condition (1). This completes the life history of the parasite within man's body. The next phase consists of its extra-corporeal existence. (6) A female mosquito sucks up some of the infected blood. (7) On arriving in the stomach of the mosquito certain of the parasites undergo changes, becoming spherical, and some of them throwing out flagella; these break away from the spheres from which they spring and accumulate round other spheres which had not thrown out flagella, and ultimately enter them. (8) These spheres (impregnated spheres) then acquire locomotive power, and, after penetrating the inner wall of the stomach, become lodged between the muscular fibres, where they (9) undergo a fresh development, and become filled with "germinal rods." (10) These germinal rods are set free in enormous numbers, and diffused by the blood throughout the insect. (11) Among the parts to which they are distributed are the poison glands connected with the proboscis. (12) When the mosquito, whose veneno-salivary glands are charged with these germinal rods, proceeds to feed on man, or, in his absence, on the particular animal that is the natural alternate host of the malaria parasite, these are inoculated along with the poison which the mosquito emits, and thus again find access to the blood, and (13) reaching the red corpuscles set up afresh the whole series of events.

Let it not be imagined that all these steps have been proved, or that anything like the whole series has been observed in regard to any one kind of bird or animal or mosquito, but recent observations render it probable that a series of events something like we have just enumerated takes place in the life history of the malaria parasite, and that the symptoms of malaria are the result of the reaction of the human body under the irritation of the invading parasite. This is the theory of to-day. As to to-morrow, who knows?

### The Stupidity of Poisoners.

In his address to the Pharmaceutical Society last week, Sir James Crichton Browne pointed out the amazing stupidity of poisoners in their choice of lethal weapons. In every age poisoning has been more or less common, but the drugs used have been but few; and even in modern times, notwithstanding the general increase of knowledge and the vast increase in the variety of poisons which are available for the destruction of life, people who use poison for murderous purposes seem driven by some mysterious insanity to cling to old methods, and to stake their necks on such easily traceable poisons as arsenic, antimony, and strychnine. From the earliest of times arsenic has been the favourite weapon of the poisoner. History abounds, no doubt, with strange stories of mysterious destructive agencies; of deaths caused, for example, by smelling a poisoned rose, or wearing a tainted ring; but when we come to investigate these cases it is seen that in regard to poisonings, as to much else, history contains many things that never happened, and that in nine cases out of ten arsenic was the material by the use of which the mediæval poisoner attained his ends. No doubt the accessibility of a poison has much to do with its selection, and one can understand in some measure the partiality of female poisoners for arsenic by remembering that this drug has long been employed in many countries as the most popular domestic exterminator of vermin, and has also been recognised as an article of toilette, so that women have been able to obtain it and explain their possession of it on these pretexts. But one would have thought that when medical men took to poisoning, they of all persons, with their knowledge of drugs and their command over them, would have been able to ring the changes in an infinite variety of ways, and to have employed alkaloidal and other organic poisons in such forms and in such combinations as to perplex the clinical observer, to baffle the pathologist, and even to elude the skill of the analyst. But it is not so. With what might almost be called infatuation they have all but invariably worked on in the old grooves, and have carefully prepared a net for their own ensnarement. The rarer and more fugacious alkaloids seem never to have been tried, while even nicotine has hardly ever been resorted to, notwithstanding the obvious difficulty which would exist in the way of proving its felonious administration in the case of a smoker, in whom any traces found might well seem but the relic of the pipe or cigarette. As to the bacteriological poisons, the toxins of disease, and other virulent microbic products, they are not as yet known to have been used at all. "For one bacteriological Mephistopheles who can possess himself of a microbic poison and use it dexterously, there will be at least a dozen despicable wretches who will take what comes to hand most readily in common life, and blunder with it egregiously." Let us hope that this is so. We must not, however, forget that in speaking as he did Sir James Crichton Browne could only be referring to cases that were found out. How far really scientific poisoning—poisoning, *i.e.*, by means of the undiscoverable poisons to which the lecturer alluded—takes place among us, there seems to be, as in fact there can be, no evidence to show. It occurs in fiction, however. Ouida depicts just such a performance, and although we do not allow that in this case fiction is even distantly founded upon fact, yet we may well admit that owing to the imitative nature of man, fact is often founded upon fiction. Every well-told story of a crime tends to reproduce itself in real life, and even Sir James Crichton Browne's address, falling upon the mad ears of some ill-balanced mind, may ere this have sown the seed of new mysterious crimes.

### The Control of Midwives.

We have received "for review" a copy of the Transactions of the Midwives' Society. This society has its home in Manchester, and the chief interest which anyone outside of Manchester is likely to feel in its transactions arises from the fact that they may fairly be imagined to indicate in some degree the aspirations of the free and independent midwives. It has perhaps been too much the habit of those who have discussed the question of the registration of midwives to assume that there are only three parties whose interests are to be considered—first, the public; second, the educated midwives who wish for registration; and third, the uneducated midwives at whose suppression the proposals for registration aim. It is well then to be reminded, as we are by the Midwives' Society, of the existence of a fourth party, whose interests will no doubt claim recognition—that, namely, of the free and independent midwives who, while objecting to the present want of system, because it leaves them exposed to the competition of the ignorant and untrained, object even more to the proposed legislation because by it they would be subjected to outside control. They claim midwifery as their own proper field, even looking upon its practice by medical men as an intrusion; yet they do not hesitate themselves to "practise" much besides midwifery. When, then, we find such a group of unqualified practitioners forming themselves into a society, and chattering about all sorts of medical topics, we cannot but feel that they thereby furnish an additional reason for some sort of legislative interference with the practice of midwifery, if only it can be insured that such legislation shall take the proper form. Hitherto interference has been asked for mostly on the ground that the lying-in woman needed protection from dirt and ignorance, but it seems abundantly clear that she will soon equally require protection from self-sufficiency, and that in any Midwives' Bill which may be proposed it will be quite as important to arrange for the control of the midwives as it will for their education. If women who practise midwifery wish to be independent of control the field is absolutely open to them, for a legal qualification is as accessible to them as it is to men. But if they refuse to undergo a complete curriculum and examination, they must in all equity submit to limitations in their practice proportionate to the limitations in their education.

